# Efficacy of the insect parasitic nematode, *Romanomermis iyengari*, for malaria vector control in Benin West Africa

**DOI:** 10.1186/1475-2875-11-S1-P5

**Published:** 2012-10-15

**Authors:** Ayaba Z Abagli, Thierv BC Alavo, Edward G Platzer

**Affiliations:** 1 Laboratoire d’Entomologie appliquée, Université d’Abomey-Calavi, BP 215 Godomey, Bénin; 2 Department of Nematology, University of California, Riverside, CA 92521-0415, USA

## Background

The intensive use of chemical insecticides against mosquitoes has led to the development of widespread insecticide resistance. Control of *Anopheles* mosquitoes in malaria-endemic areas of Sub-Saharan Africa has become increasingly difficult [1]. There is an urgent need for malaria control programs to adopt more integrated mosquito management approaches that include sustainable, non-chemical solutions. In this perspective, insect parasitic nematodes specific to mosquitoes [2,3] may be considered as alternatives, to help reduce reliance on insecticides, and concurrently help insecticide resistance management. The present work has tested the effect of the Mermithid nematode, *Romanomermis iyengari*, against *Anopheles gambiae s.s. Giles* in laboratory and field conditions in Benin, West Africa.

## Materials and methods

The nematodes *R. iyengari* were mass produced and the pre-parasitic juvenile (J2) were used in all laboratory and field experiments. Under laboratory conditions, 2 different concentrations of pre-parasitic nematodes (5 and 10 J2 per larvae) were tested against first to third instar (L1, L2 and L3) larvae of *An. gambiae.* In field, the pre-parasitic nematodes were monthly sprayed into 2 different *Anopheles* natural breeding sites in Cotonou, south Benin; 3500 and 5000 J2 per square meter of stagnant water were released, respectively in site 1 and 2.

## Results

Results indicated that in laboratory, 100% L1 larvae died within 24 hours post-infection and 100% of both L2 and L3 larvae died within 7 days post-infection, regardless of nematode concentration. In field, *Anopheles* larval density 5 days post-application decreased from 35 larvae per liter to 4 larvae, and from 17 larvae to 1, respectively in site 1 and 2. During a whole rainy season in 2011, monthly nematodes spraying resulted in suppression of larval *An. gambiae* in treated sites.

## Conclusions

The present study indicated that the Mermithid nematode *R. iyengari* is effective for malaria vector control in Benin, West Africa. *R. iyengari* mass production using local materials is easy. Integrating this nematode into *An. gambiae* management system is therefore possible.
